# Menu labelling and food choice in obese adults: a feasibility study

**DOI:** 10.1186/s40608-016-0095-3

**Published:** 2016-03-12

**Authors:** Sophie Reale, Stuart W. Flint

**Affiliations:** Academy of Sport and Physical Activity, Faculty of Health and Wellbeing, Sheffield Hallam University, Sheffield, UK; Centre for Sport and Exercise Science, Sheffield Hallam University, Sheffield, UK

**Keywords:** Obesity, Menu labelling, Food choice, Calorie, Nutrient, Energy expenditure

## Abstract

**Background:**

To date research examining the benefits of menu labelling in the UK is sparse. The aim of the present study was to examine the impact of menu labelling in a UK obese population.

**Methods:**

Using a repeated measures design, 61 patients at a tier 3 weight management service completed four questionnaires to assess their food choice (control) and behaviour change when presented with 3 menu labelling formats (calorie content; nutrient content; and energy expenditure).

**Results:**

All three forms of labelling increased participants weight control concerns compared to the control condition. There was a significant difference in content of food ordered in the three menu labelling formats compared to the control condition. The calorie condition had the largest percentage decrease in calories selected followed by energy expenditure and nutrient content. However, no difference was observed between the three conditions in the desire for menu labelling in restaurants to be introduced in the UK.

**Conclusions:**

The findings suggest that menu labelling should be enforced in the UK as it is both beneficial to promoting healthy eating and in demand. This study is the first to examine menu labelling in a UK obese population using energy expenditure equivalents to provide nutritional information.

## Background

Changes in the workforce have been observed over the past thirty years such as increased female participation and longer working hours. This has resulted in time sensitive shoppers with less complex cooking skills [1]. This combined with a rise in expendable income has led to an increase in meals eaten outside of the home. In particular, convenience foods are preferred as they require minimal preparation time and a lack of physical and mental effort [2]. The UK food and grocery convenience market has an estimated value of £46.2 billion by 2018 [3]. However, replacing home cooked meals with eating out is a concern for public health practitioners, as these foods often contain higher levels of saturated fats, which when consumed in excess, increases the risk of coronary heart disease [4].

Portion size and availability of high energy density foods has increased over the past thirty years [5], and are suggested to contribute to the obesity epidemic due to excess energy intake [6]. Evidence demonstrates that consumers and healthcare professionals with expertise in nutrition underestimate food content of meals eaten out of home [7]. This is due to poor awareness of large portion sizes and higher energy dense foods, which often leads to overconsumption and weight gain [8]. This is a concern given the associative health risks of an increased body mass index (BMI) [9] hypertension [10] and type 2 diabetes mellitus [11]. The risks of unhealthy food and drink consumption coupled with the cost of treating associative health risks, highlights the need for intervention [12].

Small step approaches are not recommended as they often steer policy development away from legislative measures, and it is these legislative measures that are suggested to have the greatest impact on public health [13]. Therefore, according to the philosophy of 'libertarian paternalism,' intervention at a national level is required to change factors in the food environment that are contributing to overconsumption and obesity prevalence. These interventions include awareness raising, discouraging overconsumption and promoting healthy eating [14]. This method ensures that an individual’s autonomy is preserved, offering an ideal solution to public policy makers.

A population based intervention that has become more common is the traffic light labelling system on all packaged foods, which is enforced by the UK Food Standards Agency (FSA) to promote healthier choices [15]. However, whilst 96 % of consumers report that they understand the labelling system (*N* = 86), only 19 % actively use it (*N* = 17) and despite using the system, no discernible effects on healthy food choice were observed [16]. Subsequently, a more uniformed food labelling system (guideline daily amount’s; GDA), was introduced to comply with the European Union’s Food Information to Consumers Regulation [17]. The inclusion of GDA’s is an approach that aims to support the population in consuming a balanced diet [18]. Studies have found a consistent link between using nutritional labels and healthy consumption [19]. However, the current labelling legislation is reported to have a minimal effect on food preference and consumption when eating out. Thus, in the absence of intervention, it is predicted that individuals will continue to consume large quantities of convenience foods whilst awareness of nutrient content remains low [20].

With the passage of The Patient Protection and Affordable Care Act in 2010, menu labelling became a federal law [21]. It mandated that restaurants in the USA retailing at 20 or more sites must provide nutrient information in the form of calories and grams in the same size and typeface as the food item [22]. By 1 December 2016, all restaurants will have nutrient information on their menus to allow consumers to make informed food choices to encourage consumption of a healthier diet. Support for the legislation has been reported from the public [23] and the restaurant industry [24]. However, the effectiveness of calorie labelling on menus to improve meal choice and thus lead to increased lower calorie meals ordered has been questioned. The findings of two systematic reviews that examined the impact of calorie labelling as an intervention to reduce calories ordered have reported that the evidence of its effectiveness is weak and inconsistent [25, 26]. For example, Schwartz et al. [26] examined studies that were predominantly conducted in fast food restaurants where consumers may not have had sufficient time to evaluate the calorific values of foods.

More promising findings have been reported in laboratory studies [27] or sit-down restaurant settings [28]. Significant reductions in calories ordered have been reported suggesting that menu labelling has the potential to impact consumer choice when time constraints are removed. Nevertheless, consumers may lack the health literacy and numeracy skills required to understand and utilise calorie information, thus causing consumer confusion [29]. A possible solution is to provide nutritional information in a more familiar and tangible fashion to enhance comprehension and increase the likelihood of improving food choices [30]. Providing calorie information as an energy expenditure equivalent may be more persuasive than calorie information. It is suggested to contextualise the information making it easier to compare menu items and thus facilitate healthier food choices [31].

Swartz and colleagues [22] conducted focus groups to assess consumer comprehension of energy expenditure labels. They reported that consumers were able to verbally interpret the energy expenditure label and as such, it prompted users to consider their food choices more often compared to current calorie labelling. Dowray et al. [32] conducted a randomised controlled trial examining the impact of energy expenditure labelling on adults’ food choice, reporting a hypothetical reduction of 100 cal ordered. It has also been reported that adolescents [33] and parent’s food choices [34] for themselves and their children, have been improved following exposure to energy expenditure labelling. However, Viera and Antonelli [34] did not compare energy expenditure labels independently (i.e., without listing calories as well) to calorie labelling or no labelling. Observations were also restricted, examining the effect of an energy expenditure labelling format on sugar sweetened beverage [33] and fast-food food choices [32]. Thus, the results are not validated or generalizable to a sit-down service restaurant menu where labelling has potential to influence food choice due to reduced time constraints.

The current study aimed to examine the effect of menu labelling on food choice in a sample of obese adults. Three menu labelling formats were compared to observe the most effective method of discouraging overconsumption and promoting healthy eating. Condition one provided calorie information, condition two provided information in grams for each of the seven categories frequently found on nutritional labels (*Fat; Saturated-Fat, Protein, Carbohydrate, of which sugars, Salt, Fibre*) and condition 3 provided a time in which an individual of 70 kg would have to sustain a moderate pace walk to achieve an energy balance if the entire meal was consumed. In line with previous research [23, 24, 27, 28, 30–34] it was hypothesised that: 1) menu labelling would encourage healthier food choices compared to no menu labelling (control); 2) the energy expenditure menu labelling format would be most effective at improving the healthiness of food choice; 3) menu labelling will encourage consumers to choose food items based on factors associated with their health and weight control; and 4) consumers would be in favour of implementing menu labelling in the UK.

## Method

### Participant and design

Following ethical approval, a convenience sample of 85 adults aged 18–78 years (Mean = 50.52, SD = 16.1) with a BMI of 30 kg.m^2^ and above (Mean = 41.17, SD = 7.44 kg∙m^2^) were recruited from a tier 3 weight management service that provides support for obese adults. An obese population was selected due to the established positive correlation between weight gain, obesity and dining out [6, 35–38]. Nine participants dropped out of the study and a further 16 were excluded as they ceased to attend the weight management service. Thus the total sample size was 61 participants comprised of 23 males and 38 females.

A randomised crossover design resulted in participants completing three experimental and a control condition (*1- calories; 2- nutrient content; 3- energy expenditure; 4- control*), in alignment with previous research examining consumer response to menu labelling [39]. Condition one provided calorie information, condition two provided information in grams for each of the seven categories frequently found on nutritional labels (*Fat; Saturated-Fat, Protein, Carbohydrate, of which sugars, Salt, Fibre*) and condition 3 provided a time in which an individual of 70 kg would have to sustain a moderate pace walk to achieve an energy balance if the entire meal was consumed. All participant’s received the control menu first followed by the experimental conditions which were presented in a randomised order. Participants attended the weight management service on four separate occasions. Participants were asked to attend at the same time of day, having consumed the same nutrients beforehand.

### Measures and materials

#### Hunger

Hunger levels were recorded in alignment with previous research [40]. A Likert scale from 1 to 10 (1 = not hungry at all; 10 = starving) was used as a controlling variable, as hunger effects appetite and thus food choice.

#### Food preference

Menu items were randomly selected from a well-known UK chain menu with nutrient data readily available. In alignment with previous research examining the effects of menu labelling [41, 42], ten meals were chosen *(large mixed grill; BBQ chicken melt; salmon salad; lasagne: jacket potato with tuna mayonnaise; 8 oz rump steak; beef burger; large cod and chips; sweet chilli egg noodles with salmon; surf and turf*). Meals were randomly selected from the website based on an average rating of 3 stars, to ensure popularity and personal preference did not affect food choice. Food items were presented randomly to prevent an order effect, as Dayan and Bar-Hillel [43] suggested that consumers are more likely to select food items at either the top or bottom of a menu. To increase ecological validity physical copies of the menus were provided. In line with Roseman et al. [44], the prices of meals were removed to avoid price influencing decisions.

#### Food Choice Questionnaire (FCQ) [45]

The FCQ is a multidimensional measure of the motives that underpin food choice, relevant to nine dimensions (*health; mood; convenience, sensory appeal; natural content; price; weight control; familiarity; ethical concern*), as these have been found to influence dietary choice. The FCQ contains 36 statements with a 4 point Likert scale from 1 to 4 (not true to very true). The Cronbach alpha scores for the subscales of the FCQ are good: 0.81 for health; 0.83 for mood; 0.84 for convenience; 0.72 for sensory appeal; 0.86 for natural content; 0.83 for price; 0.85 for weight control; 0.72 for familiarity; and 0.74 for ethical concern [45].

However, following a review of the FCQ, it was concluded that an improved version should include fewer categories and items, to increase robustness [46]. Therefore, a modified version of the FCQ was incorporated. Initially the categories, price and convenience, were removed as these were identified as not being applicable to the current study. Following a pilot study, it was determined that the dimension, ethical concern, would also be removed as no information on this category was provided. Additionally, as suggested by Fotopoulos et al. [46] the quantity of questions in the health and mood category was reduced to 3, in line with the alternative categories. This was to ensure reasoning for food choice could be examined accurately between the categories included (*natural content; weight control; sensory appeal; familiarity; health; mood*).

#### Future desire

To determine the desire for menu labelling in UK restaurants and preference among the three formats included in the current study, participants were asked to select yes or no to the question, ‘would you like to see menu labelling in this format when next dining out in the UK?’ for each of the experimental three conditions.

### Procedure

First ethical approval was obtained from Sheffield Hallam University Faculty of Health and Wellbeing Research Ethics Committee, UK. Once consent was gained, participants completed a demographic form (age, gender, BMI) and rated their current perceived hunger on a Likert scale ranging from 1 to 10. All participants completed the control condition first, followed by the three experimental conditions (calorie, nutrient and energy expenditure labelling) in a randomised order on separate visits to the weight management centre. In all conditions, participants had to initially choose a meal off a physical menu provided as if they were ordering an evening meal. This was the control menu. Participants then completed the FCQ to highlight the reasoning behind their food choice. In the control condition, the testing session was complete at this point. However, in the three experimental conditions examining menu labelling, participants estimated the content for all meals on the menu, dependant on which condition they were completing: in condition one participants estimated nutritional content in calories; in condition two participants estimated nutritional content for each individual nutrient in grams (*Fat; Saturated-Fat, Protein, Carbohydrate, of which sugars, Salt, Fibre*); and in condition three participants’ estimated the time in which a 70 kg person would have to walk to reach an energy balance having consumed that meal in its entirety (e.g., equivalent to walking 2mph for 8.5 h). Participants estimated individually for each menu item, rather than only the meal they selected, to prevent participant variation. It has been found that the larger the meal and therefore the more items it contains, the greater the discrepancy between estimated and actual values [42]. Thus, participants ordering larger meals would appear to be less knowledgeable about their chosen meal, compared to participants choosing smaller meals.

Participants then received an experimental condition menu (calorie labelling = kcal; nutrient labelling = grams; energy expenditure labelling = minutes). The extra information was displayed in a size and typeface easily readable and no smaller or larger than the font of the menu item to prevent any biasing of food choice [46]. Participants were again asked to select a meal based on the new menu provided (with objective amounts). They were instructed that they could choose the same meal as prior to the provision of information if desired. This was to assess how menu labelling affected purchasing intentions.

Completion of the FCQ was repeated to establish the reasoning for food choice following the provision of menu labelling. A final question was included, in which participants had to indicate yes or no, reflecting their desire for menu labelling in UK restaurants. On completion or withdrawal from the study, participants received a debrief letter highlighting the full purposes of the study.

### Data analysis

Repeated measures ANOVA were conducted to examine differences between the four conditions based on hunger, the six reasons for food choice measured by the FCQ, the content value of menu items selected and the preference between the three menu labelling formats. Food choice was determined by the content value of the meal item selected (e.g., calorie total in condition one) and the change in food choice was calculated by subtracting the total value selected in each of the experimental conditions from the total value selected in the control. Where significance was established pairwise comparisons were used to identify which conditions were significantly different. Differences in estimated and actual values of food and the desire for menu labelling to be introduced in UK restaurants were examined using paired t-tests. Alpha was set at .05.

## Results

### Hunger

Mean scores demonstrate that participants did not score high or low on hunger ratings (See Table 1). No significant differences were observed between mean hunger scores in each of the four conditions (*F*(3, 180) = .97, *p* > .05).

**Table 1:** Mean Likert-scale response for hunger, reason for food choice and desire for menu labelling among obese participants (*n* = 61)

Measures	Conditions			
	Control	Calorie	Nutrient	Energy expenditure
Hunger	3.16 (2.24)	3.18 (2.60)	3.56 (2.58)	2.93 (2.30)
FCQ subscales				
Natural content	1.91 (.90)	2.17 (.89)	2.21 (.91)	2.24 (.83)
Weight control	2.10 (1.08)	3.10 (.92)	3.04 (1.02)	2.98 (.94)
Sensory appeal	3.49 (.56)	3.37 (.57)	3.36 (.72)	3.22 (.73)
Familiarity	2.70 (.75)	2.57 (.75)	2.72 (.74)	2.51 (.75)
Health concern	2.03 (.80)	2.32 (.78)	2.48 (.87)	2.31 (.73)
Mood	2.53 (.83)	2.62 (.91)	2.63 (.91)	2.54 (.93)
Desire to see menu labelling		98.4 %	96.7 %	90.2 %

*FCQ* Food Choice Questionnaire based on Likert scale from 1 (not at all) to 4 (very true)

### Reason for food choice

In all three experimental conditions, there was an increase in perception of natural content, weight control, health concern and mood associated with the food choice. Whereas decreases were observed in sensory appeal and familiarity following the provision of menu labelling (See Table 1).

#### Natural content

A significant difference was identified between the control compared with the nutrient and energy expenditure labelling conditions (*F*(3180) = 3.54, *P* < .05), where participants perceived that there is more natural content in the meals when nutrient and energy expenditure information was provided in comparison to the control condition.

#### Weight concern

A significant difference was identified between the control compared to the calorie, nutrient and energy expenditure condition (*F*(3180) = 20.71, *P* < .01), where participants reported greater concern about their weight in choosing meals when presented with the calorie, nutrient and energy expenditure information compared to the control condition.

#### Taste

A significant difference was identified between the control and energy expenditure condition (*F*(3180) = 2.73, *P* < .01), where taste had a greater influence on the selected meals in the control condition compared to when energy expenditure information was presented.

#### Health

A significant difference was identified between the control and nutrient condition (*F*(3180) = 4.71, *P* < .01), where participants selected healthier meals when nutrient information was provided in comparison to the control condition.

#### Familiarity & mood state

There was no significant difference between the four conditions in relation to the familiarity of meals or the current mood state of the participant (*F*(3180) = 4.71, *P* < .01; *F*(3180) = 4.71, *P* < .01 respectively).

### Estimation versus objective amounts: the effect on food choice

In the calorie condition, participants significantly underestimated calorie content by an average of 303 kcal per meal (SD = 364.78), resulting in a mean underestimation of 28.67 % (*t*(60) = −6.50, *P* < .01). In both the nutrient and energy expenditure labelling conditions, participants overestimated content by 115.37 g (SD = 462.03) and 74.57 (SD = 502.90) minutes of exercise per meal, resulting in an overestimation of 50.89 and 6.08 % respectively. However, this overestimation was not significant (*t*(60) = 1.95, *P* > .05, *t*(60) = .27, *P* > .05 respectively).

A higher percentage of consumers underestimated content in the calorie labelling condition compared to the nutrient and energy expenditure labelling conditions (See Table 2). The calorie labelling condition also had the largest reduction in content ordered (mean = 319.17, SD = 121.27 kcal) resulting in a significant reduction of 26.03 % (SD = 28.05; See Table 3). The mean reduction was significantly larger (*P* < .05) for the participants who underestimated calorie content in comparison to those who overestimated content (See Table 2).

**Table 2:** Percentage of participants who under- and over-estimated food content and its impact on food choice in the calorie, nutrient and energy expenditure condition

Measures	Conditions		
	Calorie	Nutrient	Energy expenditure
*n* =	46	35	40
Participants who underestimated (%)	75.40	57.40	65.60
Percentage that reduced content ordered	60.90	25.70	40.00
Percentage that increased content ordered	0.00	0.00	0.00
Percentage that maintained the order	39.10	74.30	60.00
Mean content reduction (SD)	370.28 kcal* (438.91)	43.34 g (89.04)	43.61mins (123.07)
*n* =	15	26	21
Participants who overestimated (%)	24.60	42.60	34.40
Percentage that reduced content ordered	40.00	34.60	28.60
Percentage that increased content ordered	0.00	7.70	14.30
Percentage that maintained the order	60.00	57.70	57.10
Mean content reduction (SD)	158.33 kcal* (313.20)	38.15 g (75.56)	72.03 mins (115.82)

*P < .05

**Table 3:** The mean (SD) difference between the content selected in the calorie, nutrient and energy expenditure condition before and after menu labelling (*n* = 61)

	Content selected before menu labelling	Content selected after menu labelling	Magnitude of reduction	*P* value
Conditions				
Calorie	919.20 (415.57)	601.03 (254.23)	26.03 %	<.001
Nutrient	178.57 (79.67)	138.21 (57.37)	14.76 %	<.001
Energy expenditure	223.31 (125.05)	161.07 (65.27)	16.46 %	<.001

Contrastingly, the nutrient condition had the smallest percentage of consumers underestimating content (See Table 2) and the smallest reduction in content ordered following menu labelling (mean = 40.28, SD = 57.37 g) resulting in a 14.76 % (SD = 27.42) decrease in content ordered. However, there was not a significant difference in mean reduction between the participants who under and overestimated content (See Table 2).

The energy expenditure labelling condition had a reduction of 63.24 (SD = 25.05) minutes equating to a 16.46 % (SD = 32.89) decrease in content ordered following menu labelling. However, there was not a significant difference in mean reduction between the participants who under and overestimated content (See Table 2). Furthermore, no significant difference between the calorie, nutrient and energy expenditure labelling conditions were evident for reduction in content selected (*F*(2120) = 2.61, *P* > .05).

### Desire for menu labelling in the UK

Participants reported a desire for calories, nutrient and energy expenditure labelling to be included on restaurant menus (*t*(60) = 60.0, *P* < .01; *t*(60) = 42.07, *P* < .01; *t*(60) = 23.45, *P* < .01 respectively). The preferred format of menu labelling was the calorie labelling, followed by the nutrient and energy expenditure labelling (See Table 1). However, no significant difference was observed to suggest a preference for any of the three forms of labelling (*F*(2, 120) = 2.689, *P* > .05).

## Discussion

This study examined the effect of three types of menu labelling on food choice in an obese population who were autonomously adhering to a weight management programme. As such, it was expected that the participants had a strong intention to eat healthily as they were motivated to lose weight. Overall, menu labelling was an effective intervention to encourage healthy food choices, partially supporting hypothesis 1. A significant reduction in calorie content of food choice was observed post menu labelling for the calorie labelling format, when compared to the control. The results suggest that implementation of calorie information discourages consumers from choosing an unhealthy meal due to the ability to make an informed decision, in alignment with previous research [47]. The current study demonstrates that overall the largest reduction in content of meals selected was observed in the calorie labelling condition compared with the nutrient and energy expenditure conditions. In alignment with previous suggestions [48], participants appear to be more likely to actively search and use menu labelling when making food choices when calorie content is presented in comparison to other formats.

The initial food choices can be explained by nutritional awareness and the Expectancy Disconfirmation Theory (EDT) [49]. It appears that the participants were largely unaware of nutritional content of meals. A significant difference between the estimated and actual values was observed in both the calorie condition, in line with Lui et al. [50]. This suggests that without the provision of calorie information the participants were unable to make an informed food choice. The calorie condition observed the largest proportion of participants underestimating content which resulted in the largest decrease in content ordered following menu labelling. Alternatively, the nutrient condition had the largest proportion of participants overestimating content yet the most frequent amount of maintained choices and increases in content selected, in comparison to the control. Thus, hypothesis two is rejected as it was initially predicted that energy expenditure labelling would be the most effective in improving food choice. In contrast, our results demonstrate that calorie information was more beneficial in improving food choice. Thus in line with EDT, this finding may have occurred when participants were informed that their initial food choice had higher calorie content than their estimate leading to a negative disconfirmation, and consequently due to a perception that lower calories reflects a healthier meal, participants chose a lower calorie meal. In relation to those who over estimated and therefore perceived the meals to be unhealthier than in reality, it is unlikely that a different meal would be selected as this would encourage a more positive attitude towards this food choice [51], and this may explain the lesser impact of the nutrient and energy expenditure conditions in comparison.

The current study demonstrated that all three forms of labelling increased participants weight control concerns in comparison to the control condition in line with hypothesis 3. Thus, it is expected that participants weight control concerns have led to healthier food choices, and appear to reflect an effective intervention to improve food selection in obese adults attending a weight management service. However, only the nutrient condition increased participants' health concerns contradicting hypothesis 3. Thus the findings suggest that the three forms of labelling have a greater impact on weight control rather than health concerns, which may reflect the participants' attendance at a weight management service where weight is the primary focus.

Despite the observed differences, this study identified a desire for all three forms of menu labelling in line with hypothesis 4. There was no difference between the three conditions suggesting that there was no preferred form of menu labelling. Whilst there was no difference in preference, the findings suggest that the calorie labelling has a greater impact on improving food choice. This might however, reflect a greater familiarity and understanding of calorie content as suggested by Kleef et al. [52] and is included, by law, on UK food packaging. In comparison, energy expenditure is not a form of labelling employed in the UK, which may be the reason for the lesser impact compared with calorie content labelling.

This finding is vital when considering possible interventions to prevent the current rise in obesity, caused in part by unhealthy consumption. Due to environmental change, the UK population are becoming more reliant on dining out, where portion sizes are much larger than traditional home cooked meals [6]. Consumers therefore underestimate the content of meals when dining out, resulting in overconsumption of GDA's. This has led to the population gaining weight, due to minimal awareness of food content, in which they are consuming.

Menu labelling may solve this problem by increasing consumer awareness of nutritional values and content of each item on the menu. Thus, whether knowledgeable in this field or not, consumers can identify the healthier foods to make an informed decision on food choice. The current study findings have demonstrated that menu labelling may lead to a reduction in calories ordered, thus increasing the likelihood of healthy consumption. It has been suggested that as the availability of nutritional information increases, the more educated consumers will become encouraging healthy food choice and a reduction in overconsumption [53]. Menu labelling has the potential to prevent the continuity of the prevailing obesity epidemic. As such, the UK should consider implementing a law that mandates restaurants to provide nutritional information for all menu items, in particular calorie content to encourage healthy eating, prevent weight gain and reduce the risk of associated diseases (e.g., CHD). This is likely to lead to greater awareness of food content in the UK population, which is highly warranted given that no nutritional education is provided in the school education system [54]. As the population begin to utilise the information provided, it is expected that they will decrease the desire to consume unhealthy items, and the restaurant industry will need to redesign their menu to meet consumer demand [55]. Menu labelling has the potential to not only encourage healthy eating but also increase the availability of healthy foods, which is warranted in the UK's growing environment of convenience food [12].

Krieger et al. [56] suggested that the effectiveness of menu labelling may be impacted by consumers' attitudes towards their inclusion and that garnering a positive attitude is necessary. The current study demonstrated that adults attending a weight management service have a positive attitude and would welcome the inclusion of menu labelling in UK restaurants. However, this desire may reflect the current study sample and future research should establish the desire for menu labelling in other UK population groups to enhance the generalisability of findings. Future study is also warranted to counteract limitations of the current study. The sample size was small and data was collected in a meeting room thus hypothetical measures of intention were recorded rather than actual food choices [57]. It is possible that the design resulted in a bias response where participants avoided changing their initial decision due to factors such as humiliation, which could have result in an underestimation of the true effects. Additionally, repeated exposure could have impacted meal choices in subsequent visits when testing the other labelling formats. However, there was a wash out period between visits to reduce the likelihood of a learning effect. Nevertheless, the reduction in calories and nutrient content ordered were similar to the reductions observed in previous studies with a comparable design that measured actual food choice [47]. It is also unknown whether participants observed and utilised menu labelling to influence their decision. Previous research [56] has identified that consumers report seeing menu labelling but not utilising it. Thus, future research should identify participants' use of the labelling provided. One method of examining this is through the use of eye tracking software, where locations and durations of eye movements can be recorded to understand participants' natural ordering behaviour when choosing food items [58].

## Conclusion

The current study demonstrates that menu labelling may be a promising strategy for promoting healthy eating and encouraging weight loss. This feasibility study represents the first attempt at examining menu labelling in a UK obese population using energy expenditure equivalents as a method to provide nutritional information. The need to educate consumers about nutrient content is warranted given the increasing reliance on convenience food. Consumers remain unaware of larger portion sizes, which is likely to contribute to the prevalence rates of obesity and its associated diseases. By providing nutritional information in restaurants, consumers are discouraged to overconsume. Whilst there was no preference for either of the menu labelling formats, the calorie labelling condition was more effective in improving food choice. Thus, the UK government should consider menu labelling as a strategy to increase healthy food choice and consequently reduce health inequalities associated with unhealthy consumption.
